# Six Year Molars

**Published:** 1868-05

**Authors:** 


### SIX YEAR MOLARS.

Editors Register :—In the January and February numbers of your Journal, appear two articles on the care and treatment of the six year molars. That in the January number is clear and to the point. The writer proves very conclusively (and it is to be hoped that he has the majority of sensible Dentists with him,) that the indiscriminate extraction of these teeth is unscientific, and should therefore receive the severest condemnation. But while this writer and the one who follows him in the February number are satisfied that their wholesale extraction is wrong, and are very decided as to certain cases in which it would be wrong to remove them, they are not very positive as to when this course would be necessary.

The principle underlying these articles is that the six year molars should never be extracted while the jaw is growing. Now, this growth continues at least to the twenty-first year. And in connection with this principle is the kindred one of "early extraction." Do these writers assume that this "early" period is co-extensive with that during which the jaw is being developed. If they do, then they must begin very late to correct irregularities; but if they do not make this assumption, they would render their writings much more valuable to the professsion by being a little more explicit.

The question naturally arises here is it ever judicious treatment to extract any of the permanent teeth while the jaw is still in process of development? To answer this question it is necessary to consider what conditions of the mouth could suggest such a course. Setting aside the obvious one of extensive decay, irregularity, in a multitude of cases points to extraction.

It is a matter of fact that deformity occurs in the mouth oftener than in any other part of the body. It is a common occurrence to observe in slender females with thin, small jawbones, teeth suitable for the large-boned man, and to observe in the mouth of a full sized, massive jawed man, teeth small and white like those appropriate to the mouth of a delicate lady. This is a result of the hereditary principle and of marriages between persons badly matched physically. A large-boned man, one with large jaws and teeth to correspond, takes to wife a lady small, and delicate, with small jaws and small teeth. Both according to their size are perfectly proportioned. Their daughter inherits the general physique of the mother, but the teeth of the father. In the thin small jaw are found not the small white teeth, but teeth suitable for a jaw twice as large.

This is a monstrosity in a small way, and in most cases ends in such irregularity as destroys the perfect symmetry of the face if not attended to in due time. It is useless to say, let the teeth remain, the jaw will accommodate itself to them; it will not, at least not by enlarging itself so as to make teeth and jaw more nearly proportioned to each other. Such enlargement may be produced artificially, but at the risk of a more marked deformity than previously existed. What Dentist has not seen large teeth so crowded in a small jaw that the centrals were forced to project from the alveolus beyond the lip, or at such an angle that the lip was permanently held out of place, giving an unsymmetrical appearance to the mouth. And such a condition might have been prevented by timely treatment. When the teeth are fully developed is not the time for this, though even then something may be done by artificial aid. It is while the jaw and the teeth are growing that nature can best be assisted to remedy the defect. Here then is a case where extraction is clearly justifiable, even when the teeth are all sound. Why spare the six year molars in preference to any other teeth. If either of the bicuspids be decayed of course its removal would be indicated rather than that of the molar; but if the bicuspids are sound then the first molar should be sacrificed. And why? Because it is erupted at an earlier period, when the vital powers are not so strong as when the other molars and the bicuspids are erupted, and it is therefore, other things being equal, more likely to succumb, before them, to the action of injurious agents.

While all honest Dentists should reprobate the wholesale sacrifice of the first permanent molars, they should not perhaps rush into the other extreme of never extracting them at all until the jaw be fully developed, whether they be extensively decayed or not, and whether or not the health may be affected thereby.

There is no doubt that empirics are reckless about this class of teeth, and that many honest Dentists sacrifice them unthinkingly. But this malpractice can only be corrected by *proving* to the wrong-doers that they are in error. It can never be exorcised out of the profession by parson, book and bell.

